# Supplementary material for: Systematic identification of Y-chromosome gene functions in mouse spermatogenesis
Source: Science. Author manuscript; Available in PMC 2025 Feb 9. (PMC7617377; doi:10.1126/science.ads6495)
Supplement: Supplementary Material [file EMS202884-supplement-Supplementary_Material.pdf]

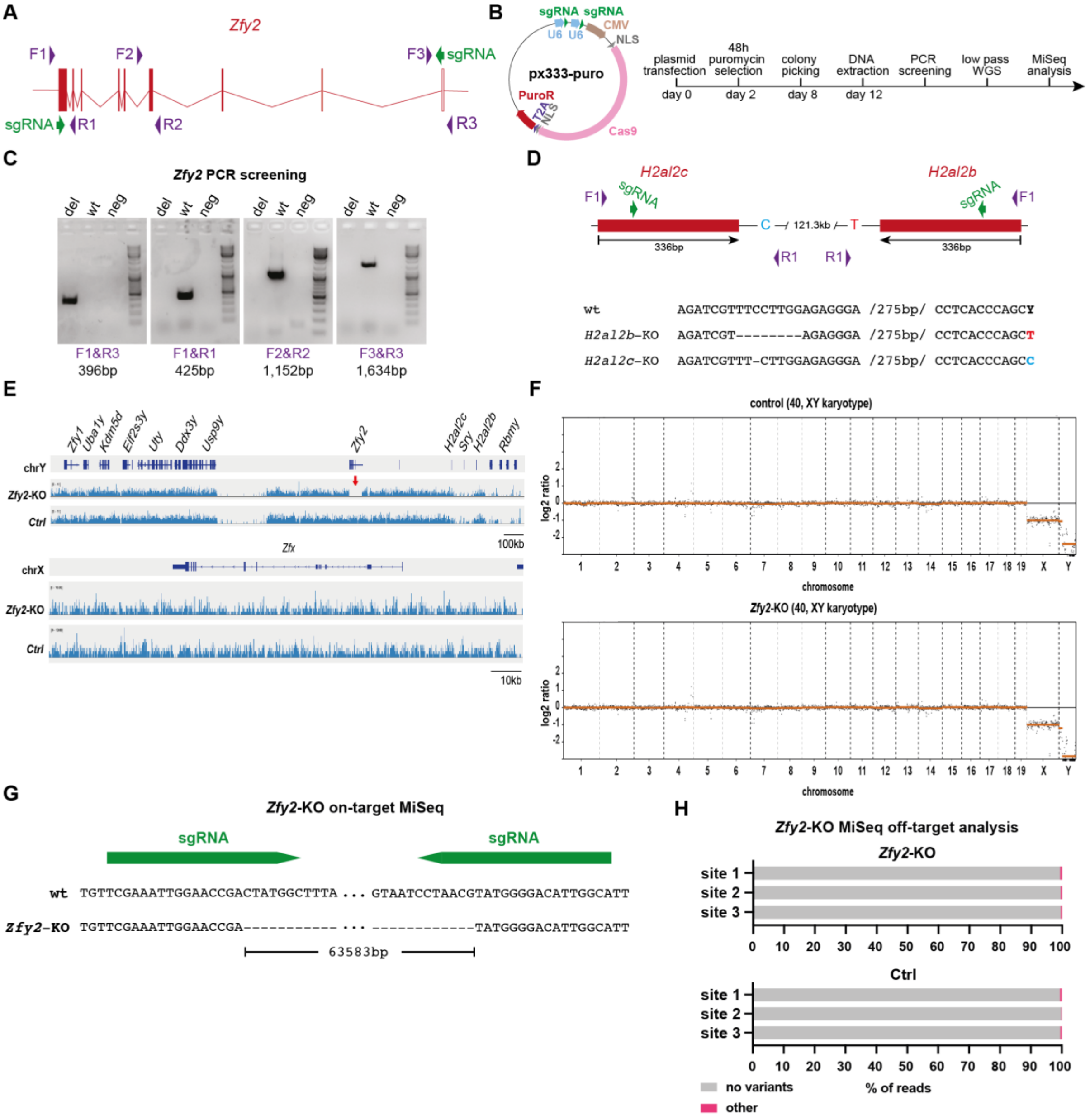


**Fig. S1: Y-gene targeting and screening pipeline in mouse embryonic stem cells.**
(**A**) The *Zfy2* locus showing the location of sgRNAs and primers as an example of single gene targeting strategy. (**B**) Map of the “all in one” px333-puro plasmid and timeline of mESC targeting. (**C**) PCR screening approach for *Zfy2* targeting. Deletion, upstream boundary, exonic, and downstream boundary PCRs are shown (see panel **A**, for reference primers) for a *Zfy2*-KO clone, wildtype control, and negative control (water). (**D**)The duplicated *H2al2c* and *H2al2b* loci showing the sgRNA, primers, and SNP used for targeting and sequencing. MiSeq reads of a clone with successful editing in both copies are shown. (**E**) Low pass whole-genome sequencing (WGS) reads for *Zfy2*-KO and control clones mapped to the short arm of the Y chromosome and *Zfx*. Red arrow indicates successful deletion of *Zfy2*. (**F**) Chromosomal copy number analysis for a control and *Zfy2*-KO clone showing normal karyotype. Analysis performed using the QDNAseq R package on a low pass WGS dataset. (**G**) MiSeq reads of a *Zfy2*-KO clone showing a 63,583 bp deletion of the *Zfy2* locus. (**H**) Analysis of MiSeq reads of a *Zfy2*-

KO and control wildtype clone at three potential off-target sites. Analysis performed with the CrispRVariants R package.

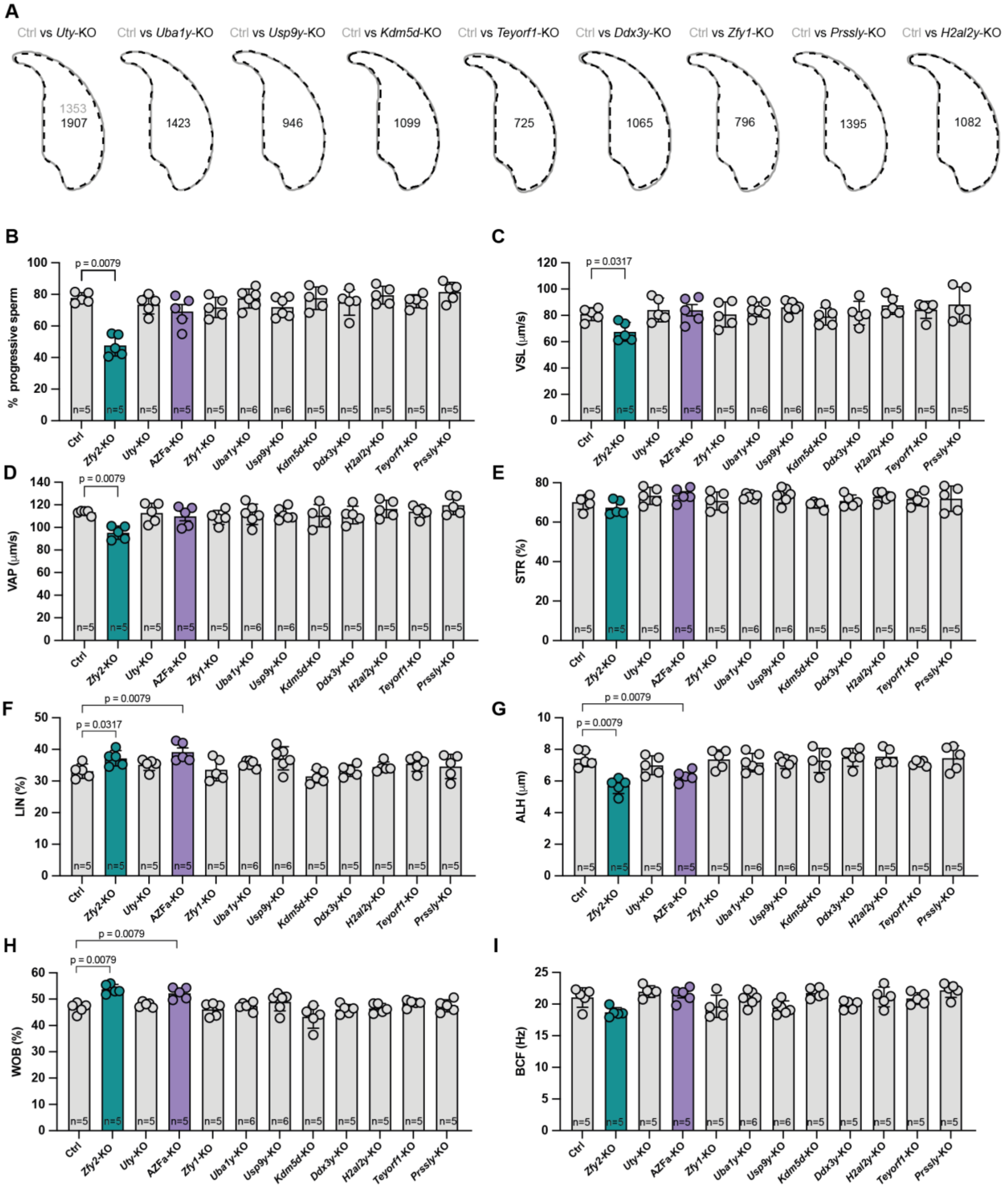


**Fig. S2: Sperm head morphology and motility analysis in Y-gene deletants.**

(**A**) Average sperm head profile compared between control and Y deletants showing no abnormalities. The number of sperm heads used to build the consensus morphologies are indicated. (**B**) Average percentage of progressive sperm for control and Y deletant males. (**C**) Average straight-line velocity (VSL) of sperm from control and Y deletant males. (**D**) Average path velocity (VAP) of sperm from control and Y deletant males. (**E**) Mean straightness (STR) of sperm from control and Y deletant males. (**F**) Mean linearity (LIN) of sperm from control and Y deletant males. (**G**) Mean lateral head displacement (ALH) of sperm from control and Y deletant males. (**H**) Mean wobble (WOB) of sperm

from control and Y deletant males. (**I**) Mean beat cross frequency (BCF) of sperm from control and Y deletant males. All n = number of males. All error bars = standard error of the mean. All statistical analysis by Mann Whitney test.

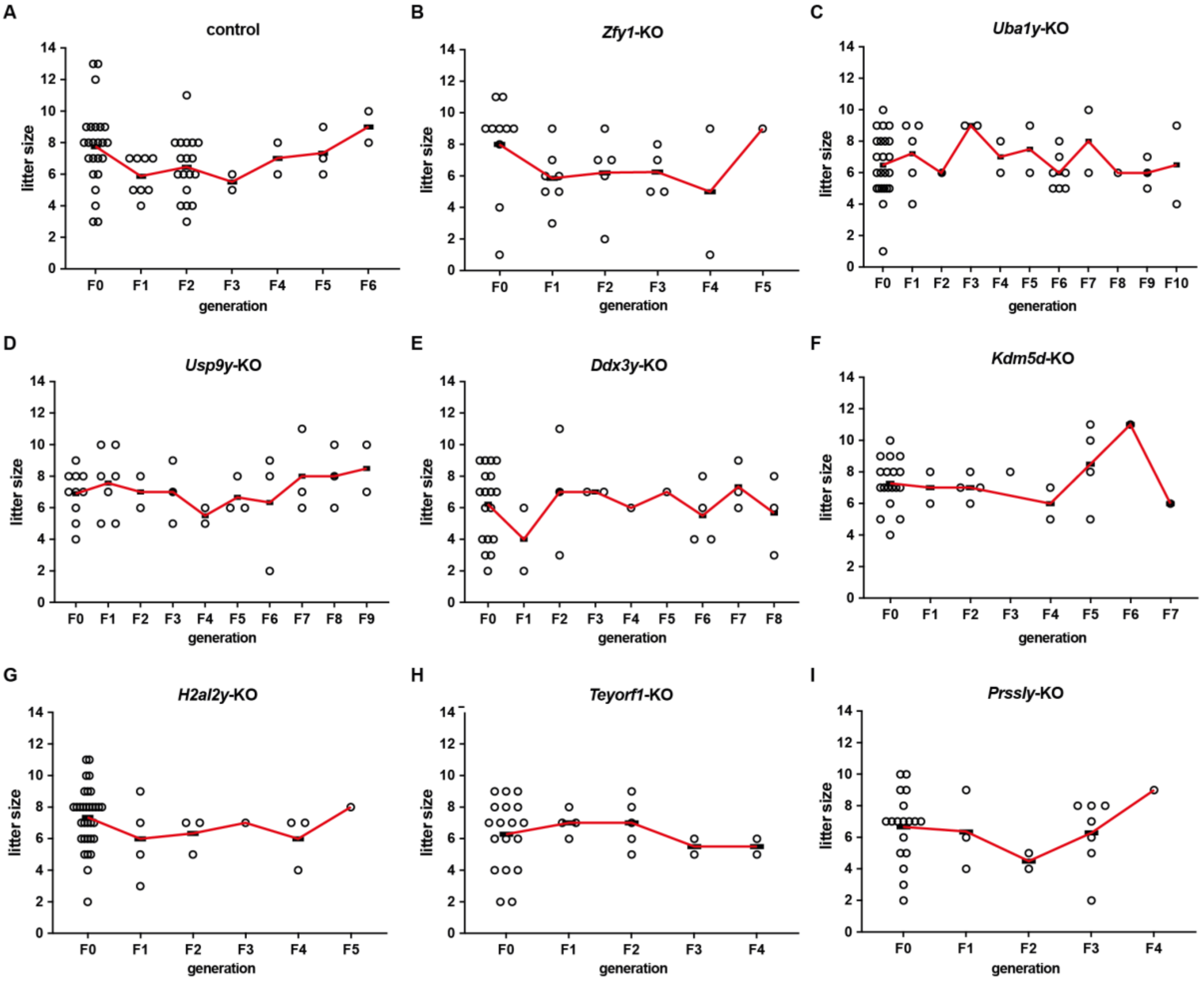


**Fig. S3: Litter sizes across filial generations for Y-deletants with no spermatogenic abnormalities.** (**A-I**) Number of pups born per litter across generations of control (A), *Zfy1*-KO (B), *Uba1y*-KO (C), *Usp9y*-KO (D), *Ddx3y*-KO (E), *Kdm5d*-KO (F), *H2al2y*-KO (G), *Teyorf1*-KO (H), and *Prssly*-KO (I). Mean litter sizes across generations are linked by a red line.

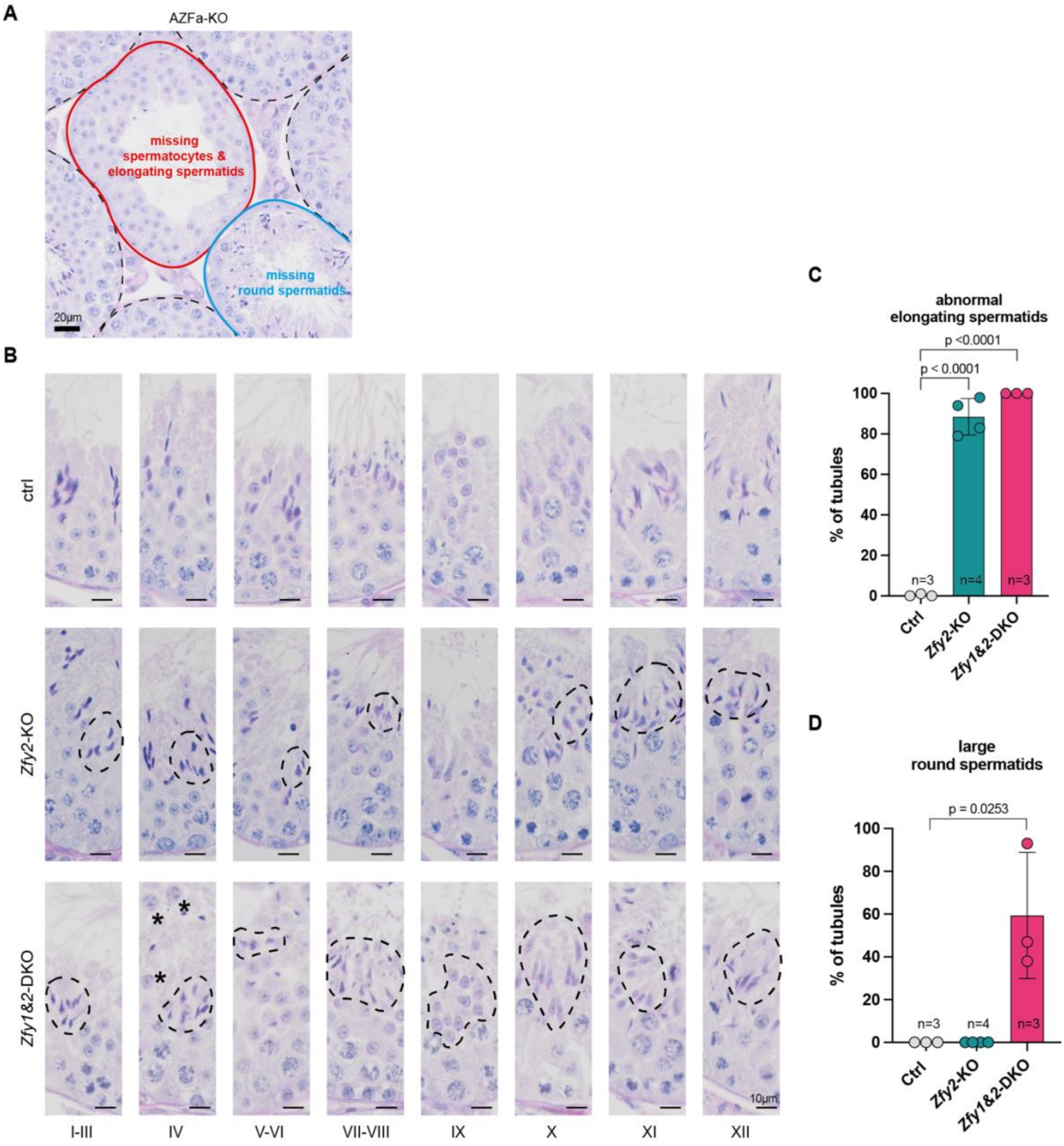


**Fig. S4: Abnormal seminiferous cycle and tubules in AZFa and *Zfy* deletant models.** (**A**) PAS-stained testis section of AZFa-KO showing tubules with missing generations (red and blue outline) compared to normal tubules (black dashed outline). Scale bars = 20 µm. (**B**) PAS-stained testis sections from control, *Zfy2*-KO, and *Zfy1&2*-DKO at different stages of the seminiferous cycle. Asterisks mark “large”, likely diploid, round spermatids. Example elongating spermatids showing abnormal morphology are encircled by dotted lines. Scale bars = 10 µm. (**C**) Percentage of seminiferous tubules containing more than five elongating spermatids with abnormal morphology in controls, *Zfy2*-KO, and *Zfy1&2*-DKO. (**D**) Percentage of seminiferous tubules containing more than five “large”, likely diploid, round spermatids in controls, *Zfy2*-KO, and *Zfy1&2*-DKO. All n = number of males. All error bars = standard deviation. All statistical analysis by unpaired two tailed t test.

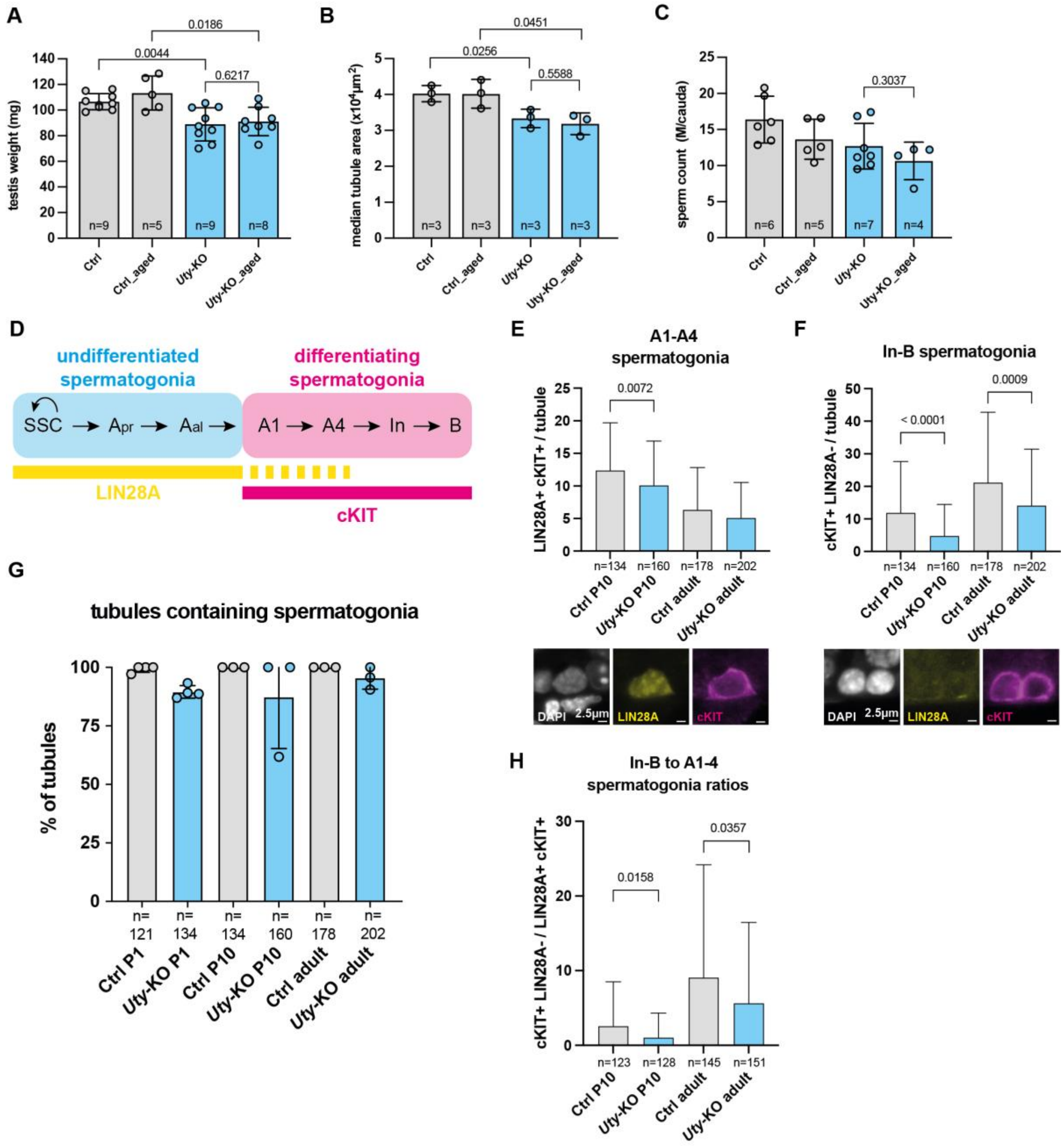


**Fig. S5: Characterization of *Uty*-KO spermatogenic defects in juvenile, adult, and aged males.** (**A**) Average testis weight of adult (13-15 weeks) and aged (43 weeks) controls and *Uty*-KO. n = number of males. Statistical analysis by Mann Whitney test. (**B**) Median seminiferous tubule area of adult and aged controls and *Uty*-KO control. n = number of replicate males. At least 40 tubules were counted per replicate. Statistical analysis by unpaired two tailed t test. (**C**) Average cauda epididymis sperm count per animal for adult and aged controls and *Uty*-KO. n = number of males. Statistical analysis by unpaired two tailed t test. (**D**) Diagram showing the developmental progression of spermatogonial cells, and when LIN28A and cKIT markers are detected. (**E**) Quantification of A1-A4 spermatogonia per tubule in P10 and adult controls compared to *Uty*-KO mice, identified by positive immunostaining for LIN28A and cKIT. n = number of tubules across three biological replicates. Statistical analysis by Mann Whitney

test. (**F**) Quantification of In-B spermatogonia per tubule in P10 and adult controls compared to *Uty*-KO mice, identified by positive cKIT signal and negative LIN28A staining. n = number of tubules across three biological replicates. Statistical analysis by Mann Whitney test. (**G**) Quantification of the percentage of tubules containing at least one spermatogonial cell. Spermatogonial cells were identified either as LIN28A, cKIT or double positives. (**H**) Quantification of In-B to A1-A4 spermatogonia ratios between controls and *Uty*-KO in P10 and adult tubules. n = number of tubules. Statistical analysis by Mann Whitney test. All error bars = standard deviation. Scale bars = 2.5 μm.

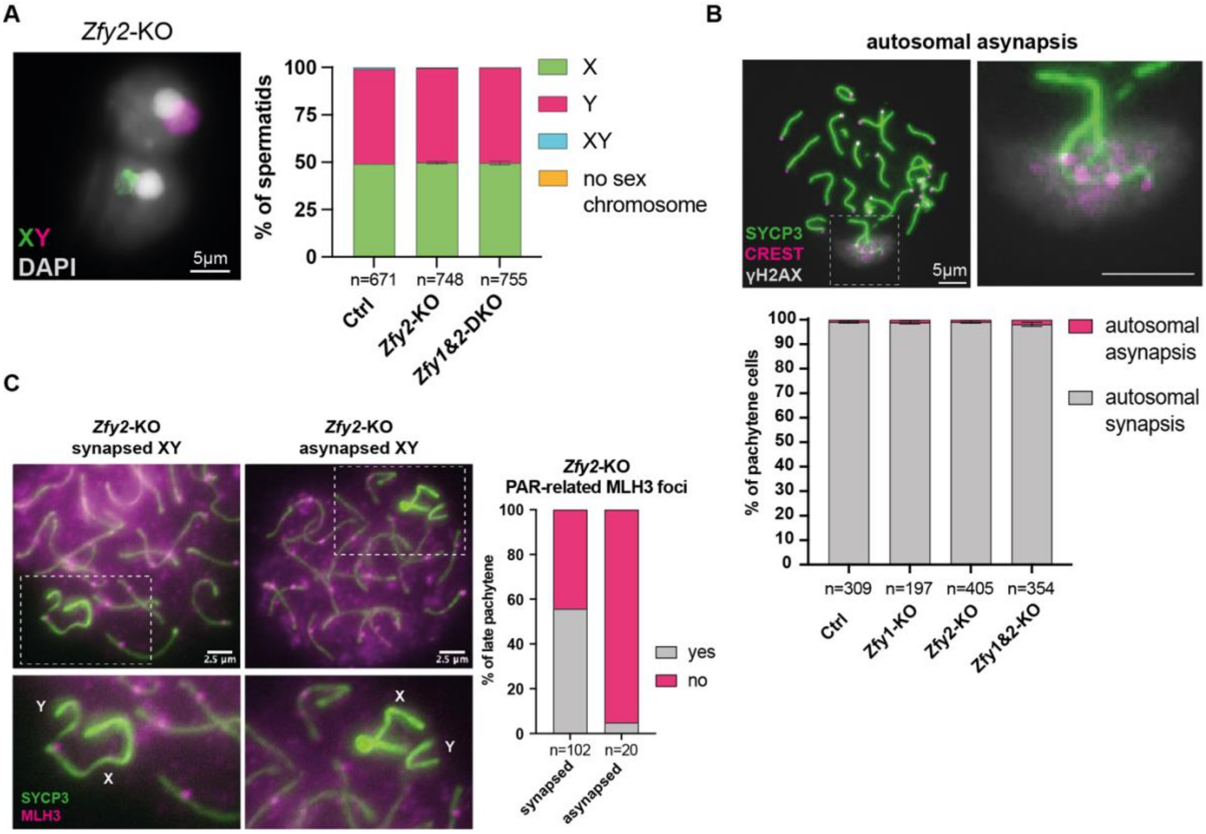


**Fig. S6: Characterization of *Zfy* functions in chromosome synapsis.**

(**A**) X and Y chromosome painting of round spermatids to quantify the percentage of spermatids bearing X, Y, XY, or no sex chromosomes in controls, *Zfy2*-KO and *Zfy1&2*-DKO. Scale bar = 5 µm. Error bars = range. (**B**) Pachytene spermatocytes immunostained for SYCP3 (green), CREST (magenta), and γH2AX (grey) to quantify autosomal asynapsis in control and *Zfy* deletants. Inset shows an asynapsed autosome pair. Scale bars = 5 µm. n = number of pachytene counted across three biological replicates. Error bars = standard error of the mean. (**C**) Late pachytene spermatocytes of *Zfy2*-KO immunostained for SYCP3 (green) and MLH3 (magenta) to quantify crossovers in sex chromosomes. Scale bars = 2.5 µm. n = number of cells counted across two biological replicates.

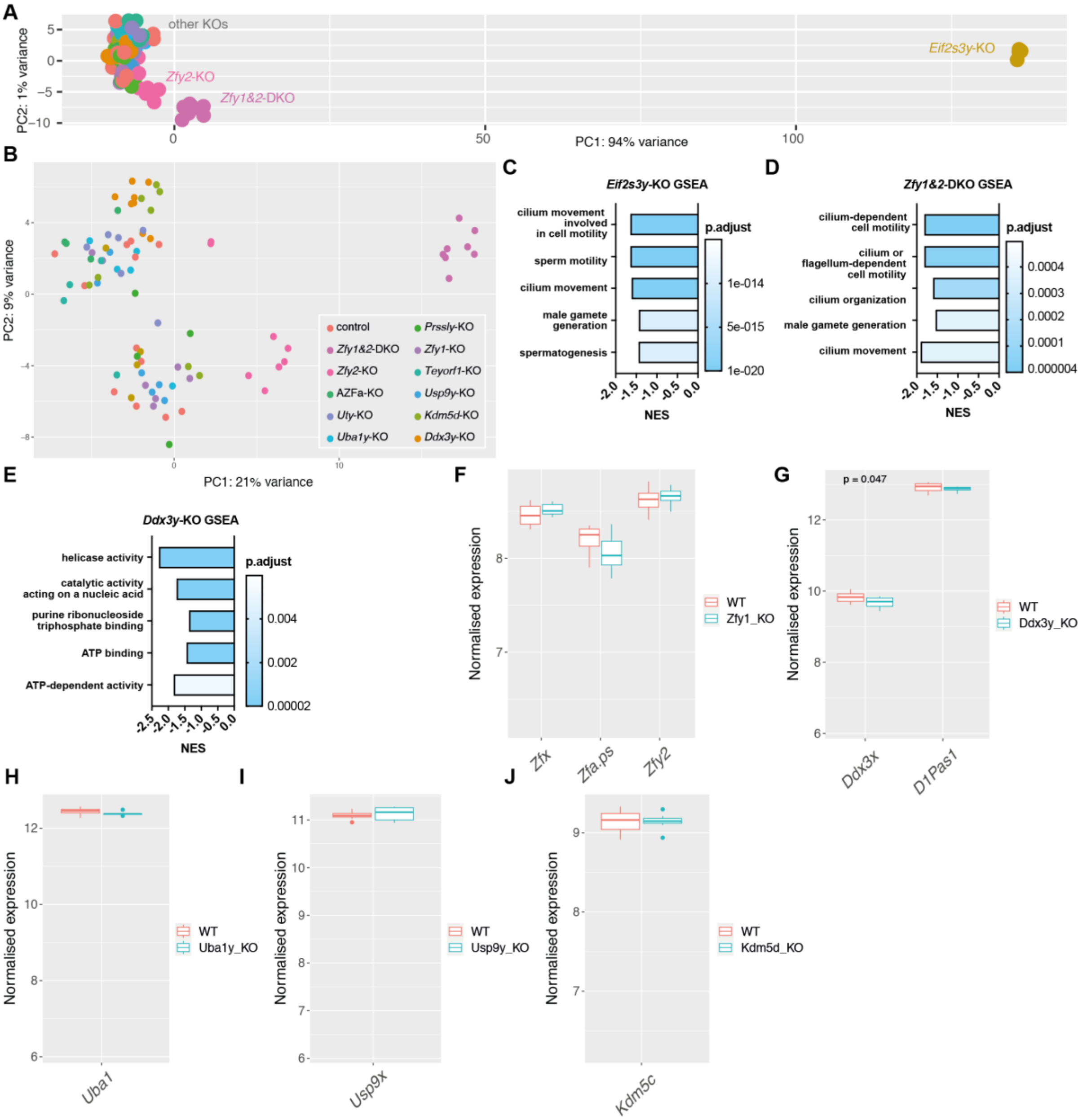


**Fig. S7: Characterizing the effects of Y deletions on the testis bulk transcriptome.** (**A**) Principal component analysis (PCA) of testis bulk RNAseq for controls and the 13 Y-deletants. (**B**) PCA excluding *Eif2s3y*-KO samples. (**C**) Gene set enrichment analysis (GSEA) showing the top five downregulated gene ontology terms based on adjusted p-values in bulk samples of *Eif2s3y*-KO, for (**D**) *Zfy1&2*-DKO, and (**E**) *Ddx3y*-KO. The normalized enrichment score (NES) is plotted. (**F**) Normalized expression of the *Zfy1* homologues, *Zfx* (X-encoded), *Zfa.ps* (autosomal) and *Zfy2*, in control and *Zfy1*-KO testis bulk RNAseq. (**G**) Normalized expression of *Ddx3y*'s homologues *Ddx3x* (X-encoded) and *D1Pas1* (autosomal) in control and *Ddx3y*-KO testis bulk RNAseq. (**H**) Normalized *Uba1* expression (X homologue of *Uba1y*) in control and *Uba1y*-KO testis bulk RNAseq. (**I**) Normalized *Usp9x* expression (X homologue of *Usp9y*) in control and *Usp9y*-KO testis bulk RNAseq. (**J**) Normalized *Kdm5c* expression (X homologue of *Kdm5d*) in control and *Kdm5d*-KO testis bulk RNAseq. Statistical analysis by unpaired two-sided t test with only significant (<0.05) p values shown.

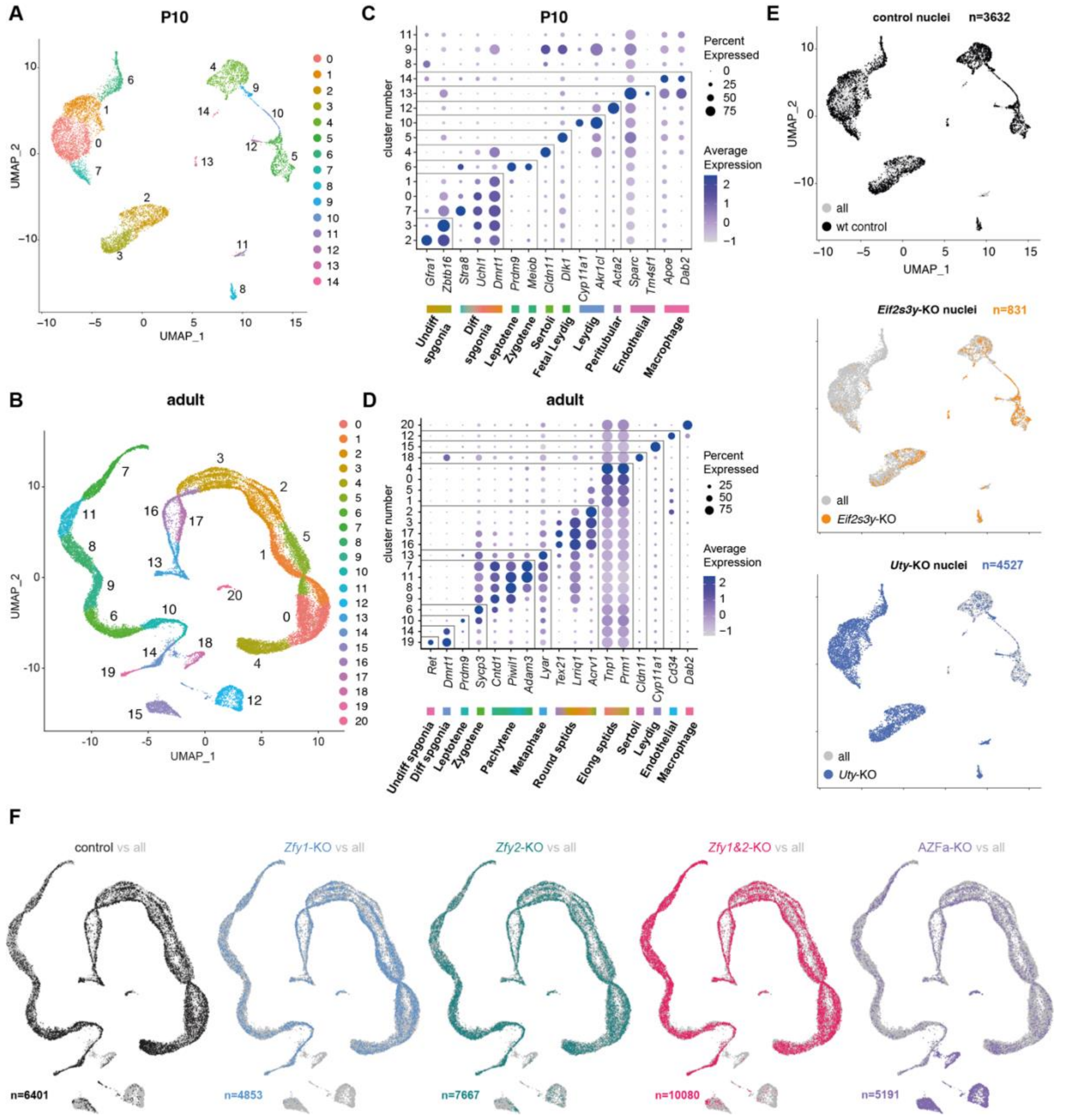


**Fig. S8: Clustering and annotation of 10x single nuclei RNAseq datasets.**

(**A**) Integrated UMAP of P10 testes clustered at the 0.5 resolution showing 15 clusters. (**B**)Integrated UMAP of adult testes clustered at the 0.5 resolution showing 21 clusters. (**C**) Dot plot used for P10 cell type assignment based on marker gene expression. Clusters 8, 9, and 11 do not have a clear signature and were annotated as "unknown". (**D**) Dot plot used for adult cell type assignment based on marker gene expression. (**E-F**) UMAPs showing the contribution of each genotype for P10 (E) and adult (F). The number of nuclei is shown for each genotype.

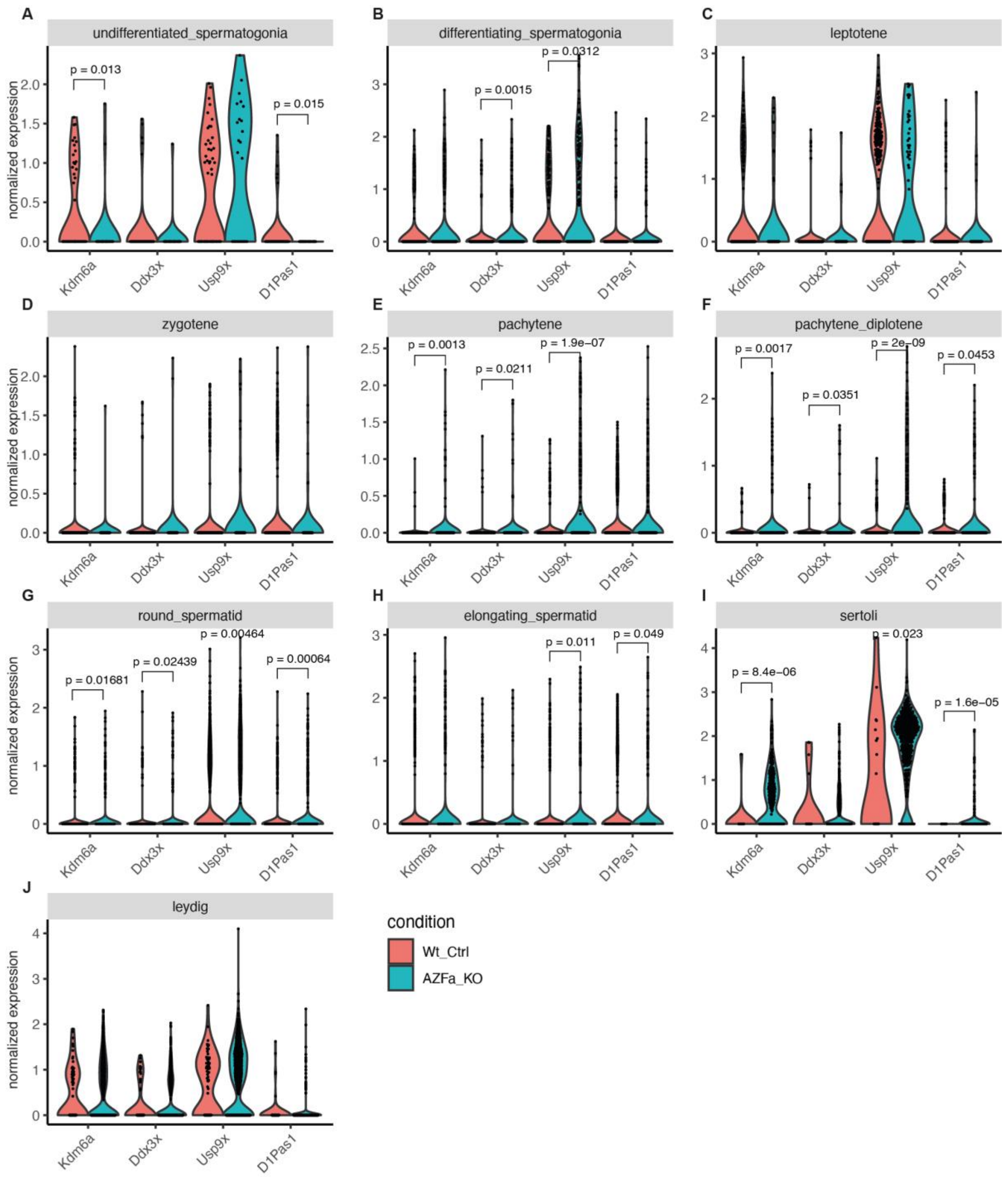


**Fig. S9: Expression of AZFa homologues in spermatogenic cell types.**

(**A-J**) Violin plots showing the normalized expression of *Kdm6a* (also known as *Utx*), *Ddx3x*, *Usp9x* and *D1Pas1* in controls and AZFa-KO clusters for undifferentiated spermatogonia (A), differentiating spermatogonia (B), leptotene (C), zygotene (D), pachytene (E), pachytene and diplotene (F), round spermatid (G), elongating spermatid (H), Sertoli (I), and Leydig (J) cell types. Dots represent expression value in individual cells. p values were calculated using an unpaired two sided t test and plotted in the graph if significant (<0.05).

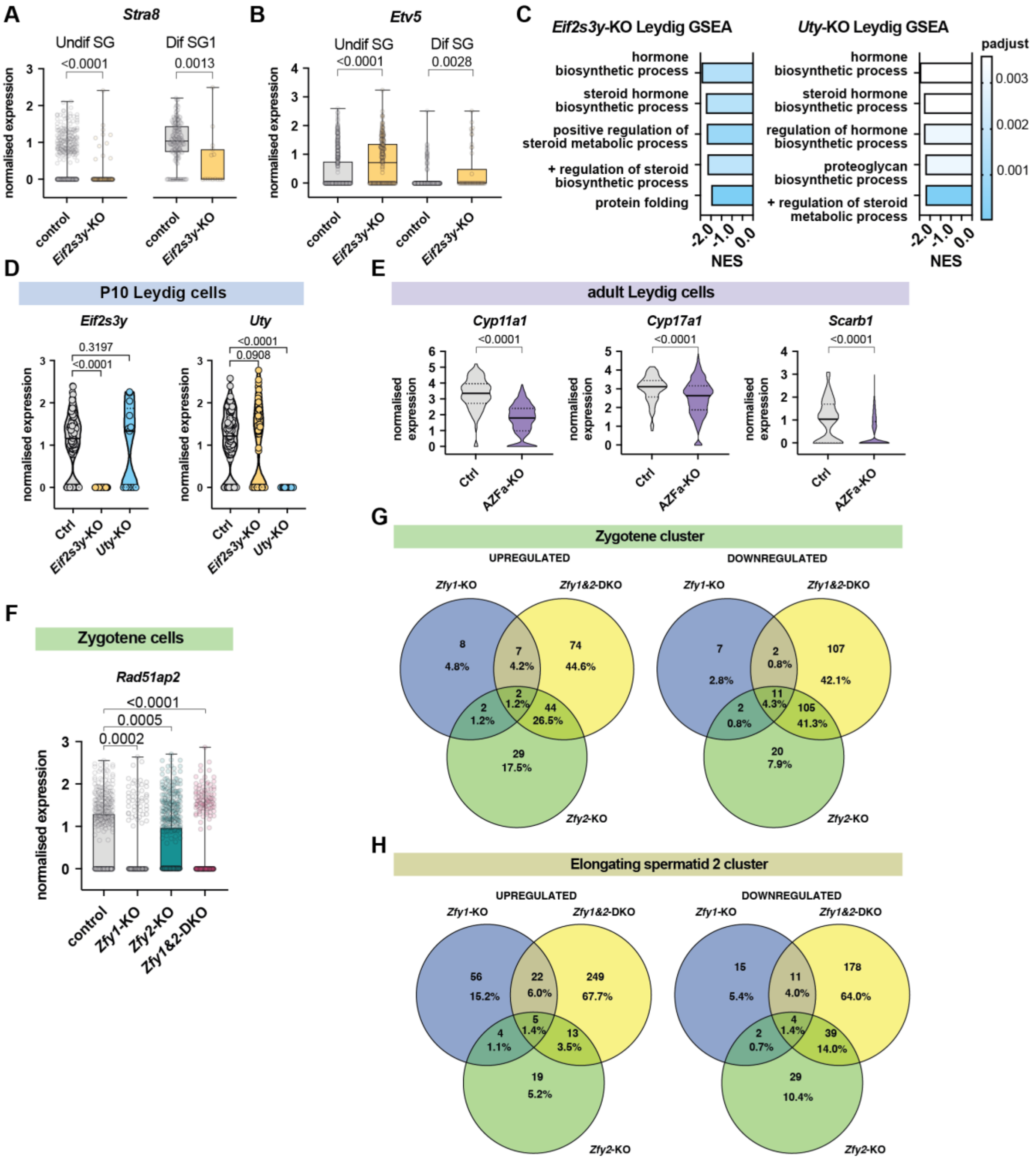


**Fig. S10: Gene expression analyses of 10x single nuclei RNAseq datasets.**

(**A**) Normalized expression of the differentiation marker *Stra8* in merged undifferentiated (Undif SG) and differentiating spermatogonia 1 P10 clusters (Dif SG1) for *Eif2s3y*-KO and control. (**B**) Normalized expression of the progenitor marker *Etv5* in merged undifferentiated (Undif SG) and merged differentiating spermatogonia P10 clusters (Dif SG) for *Eif2s3y*-KO and control. (**C**) GSEA analysis in P10 Leydig cells of *Eif2s3y*-KO and *Uty*-KO showing the top five downregulated gene ontology terms based on normalized enrichment score (NES). (**D**) Expression of *Eif2s3y* and *Uty* in control, *Eif2s3y*-KO, and *Uty*-KO P10 Leydig cells. (**E**) Normalized expression of genes involved in the steroidogenesis

pathway in adult control and AZFa-KO adult Leydig cells. (**F**) Normalized expression of the recombinase *Rad51ap2* in control, *Zfy1*-KO, *Zfy2*-KO, and *Zfy1&2*-DKO adult zygotene cells. (**G-H**) Overlap in DE genes between *Zfy1*, *Zfy2,* and *Zfy1&2* KOs in (G) zygotene and (H) elongating spermatid clusters. All p values calculated by Kolmogorov-Smirnov test. For box plots, center line is the median; box limits, 25th and 75th percentile; whiskers, minimum to maximum; points, outliers.

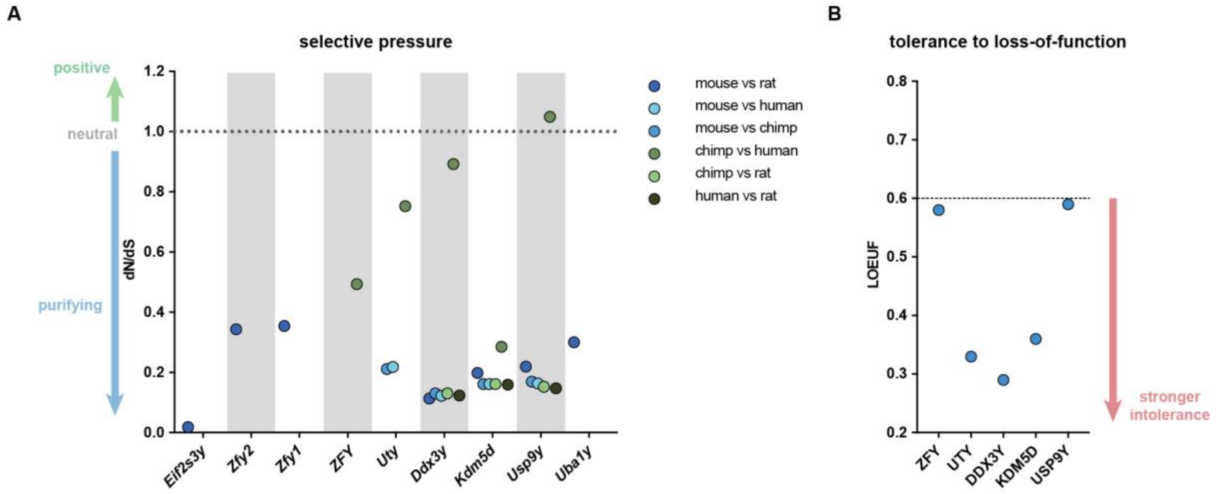


**Fig. S11: Selective pressure on broadly conserved mammalian Y genes.**

(**A**) Ratio of nonsynonymous to synonymous substitution rates (dN/dS) in broadly conserved Y genes between mouse, rat, chimp and human Y genes. Ratios below 1 indicate purifying selection. (**B**) Loss-of-function Observed/Expected Upper-bound Fraction (LOEUF) score plotted from the gnomAD v2.1.1 database. Scores below 0.6 indicate high intolerance to loss-of-function variation.
